# Deciphering the Effects and Mechanisms of Yi-Fei-San-Jie-pill on Non-Small Cell Lung Cancer With Integrating Network Target Analysis and Experimental Validation

**DOI:** 10.3389/fphar.2022.851554

**Published:** 2022-05-11

**Authors:** Hongxing Yang, Qiuyan Guo, Jianbin Wu, Lixia Zhong, Lingling Sun, Wei Liu, Jigang Wang, Lizhu Lin

**Affiliations:** ^1^ Department of Oncology, The First Affiliated Hospital of Guangzhou University of Chinese Medicine, Guangzhou, China; ^2^ Artemisinin Research Center, and Institute of Chinese Materia Medica, China Academy of Chinese Medical Sciences, Beijing, China; ^3^ Central People’s Hospital of Zhanjiang, Zhanjiang, China; ^4^ Department of Oncology, the Affiliated Hospital of Southwest Medical University, Luzhou, China

**Keywords:** non-small cell lung cancer, Yi-Fei-San-Jie-pill, orthotopic model, apoptosis, autophagy, immune infiltration

## Abstract

Non-small cell lung cancer (NSCLC), which accounts for 85% of lung cancer cases, calls for better therapy. Yi-Fei-San-Jie-pill (YFSJ), a well-applicated traditional Chinese medicine formula, was reported to be effective in the treatment of NSCLC. However, its anti-tumor mechanism still needs to be fully elucidated. Herein, a reliable preclinical orthotopic but not subcutaneous model of NSCLC in mice was established to evaluate the anti-cancer properties and further validate the mechanisms of YFSJ. A bioinformatic analysis was executed to identify the potential targets and key pathways of YFSJ on NSCLC. In detail, the anti-tumor effect of YFSJ and the autophagy inhibitor 3-MA was evaluated according to the tumor fluorescence value and comparison of different groups’ survival times. As a result, YFSJ markedly decreased tumor size and prolonged survival time in contrast with those in the orthotopic model group (*p* < 0.05), and it also significantly regulated the protein expression levels of apoptosis- and autophagy-related proteins. In conclusion, this study provides convincing evidence that YFSJ could inhibit the growth of tumors and prolong the survival time of tumor-bearing mice based on the NSCLC orthotopic model, and its anti-tumor effect was closely associated with the promotion of apoptosis and interference of autophagy coupled with regulation of immune infiltration.

## Introduction

Lung cancer has ranked as the world's first malignant tumor in recent decades, with non-small cell lung cancer (NSCLC) being the most common pathological type. The 5-year survival rate of NSCLC is merely 15–16% (Tfayli et al., 2021). Chemotherapy and radiotherapy, ranking first-class treatments of NSCLC, however, are prone to cause serious side effects and acquired resistance. While, targeted therapy reaches a potent response and definite effect on NSCLC driven by specific gene mutations, the acquired resistance often appears within 1 year (Hirsch et al., 2016). Therefore, the challenge of drug resistance needs to be solved urgently. Immunotherapy pushes lung cancer treatment into a broad new era and has brought great confidence to patients whereas its objective response rate in NSCLC is not as high as expected, and even about 20% of progressed lung cancer patients have severe side effects such as super-progression (Kim et al., 2019). Hence, it is necessary to control its side effects and improve its response rate. Previous studies (Chang et al., 2013; Lou et al., 2016) have shown that traditional Chinese medicine (TCM) treatment of cancer plays an important role in alleviating the side effects of chemotherapy and radiotherapy, decreasing acquired resistance, improving patients’ life quality, and prolonging survival time. Yi-Fei-San-Jie-pill (YFSJ), a long-time applied formula by our research team targeting replenishing Qi and removing phlegm, has been made into hospital preparation and gained a patent (201710010908.4). Some clinical studies (Zhou DH. et al., 2005; Zhou D. et al., 2005) have demonstrated that YFSJ stabilizes tumors, prolongs survival, and improves the life quality of lung cancer patients, but its anti-cancer mechanisms are still not fully elucidated.

Bioinformatics has played an increasingly important role in the era of multi-omics and has a wide range of applications in the interpretation of complicated diseases such as cancer that are considered a systems disease and the discovery or mechanistic elucidation of drugs (Yildirim et al., 2007). Network pharmacology, a classic methodological example of bioinformatics based on systems biology (Hopkins, 2007), has strong explanatory power in the demonstration of TCM pharmacology. The commonly used methods of network pharmacology include CIPHER, DMIM network pharmacology algorithm, etc. (Li and Zhang, 2013). Given that there have been successful cases of network pharmacology in the discovery of kinds of drugs (Hopkins, 2008) and the reverse of side effects (Brown et al., 2015), and this study was based on the study of TCM formula. It was rational and necessary to use network pharmacology to explore the potential mechanism of YFSJ and then verifying it with wet experiments, so as to be more convincing in the demonstration of the conclusion (Tang and Aittokallio, 2014). Furthermore, bioinformatics coupled with an orthotopic but not subcutaneous model of lung cancer can be more attractive and enlightening, for the orthotopic model is more faithful to cancer pathology (Weiss et al., 2015).

**FIGURE 1:**
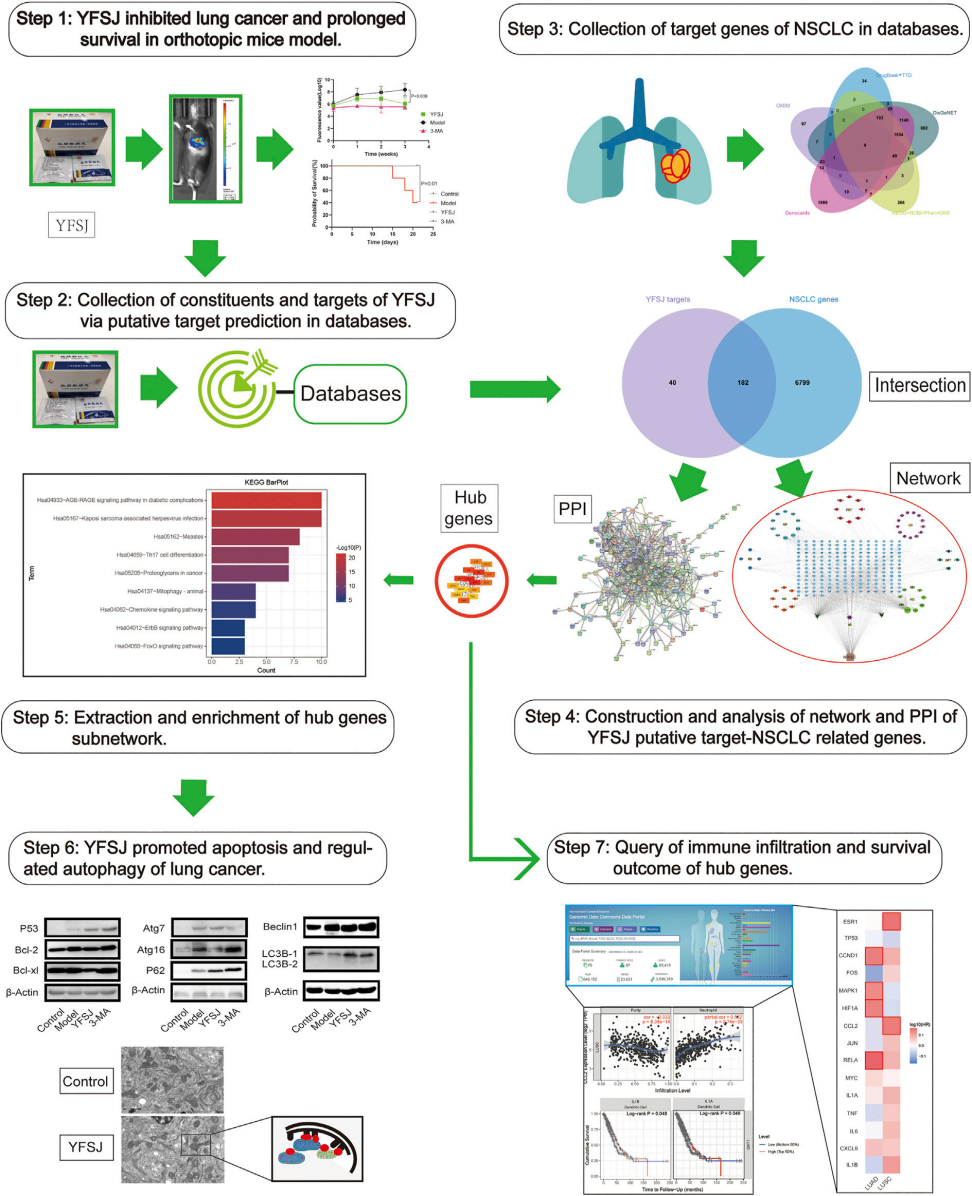
The graphical abstract of the systematic strategies for the determination of the pharmacological mechanisms of YFSJ on NSCLC.

Therefore, bioinformatic investigation coupled with the experimental exploration based on an orthotopic model of lung cancer in mice was performed in this study (Figure 1). The effect of tumor inhibition was evaluated by comparing the fluorescence values captured by using the small animal live imaging instrument, and the survival time of different groups was calculated by the statistical method of survival analysis. The key targets of YFSJ were extracted via the MCC algorithm of the Cytohubba plug-in of Cytoscape and further analyzed via GO enrichment and KEGG enrichment. Apoptosis and au(mi)tophagy were indicated as the most related two biological processes of NSCLC. Western blot was performed to investigate the molecular mechanism based on the bioinformatic analysis results by evaluating the expression levels of the apoptosis-related proteins and the autophagy-related proteins. We discovered that the tumor size of mice that accepted YFSJ for 3 weeks was markedly decreased in contrast with those in the orthotopic model group (*p* < 0.05). And YFSJ dramatically prolonged the survival time of tumor-bearing mice (*p* < 0.05). The key targets of YFSJ acting on NSCLC were closely enriched in apoptosis, autophagy, and immune-related biological processes were also implicated in.

## Material

6–8 weeks old SPF-grade female C57BL/6N mice (purchased from Beijing Wei Tong Li Hua Laboratory Animal Technology, SCXK (Beijing) 2016-0006), Matrigel (BD, NO 356234), LLC-luc cells (purchased from Shanghai Zhong Qiao Xin Zhou Biotechnology Co., Ltd. (LZQ0009)), shaver (Codos, CP-8000), disposable sterile insulin syringe (BD, 328421), disposable sterile syringe (BD, 300841), D-luciferin (Potassium Salt, Gold Bio Technology, LUCK-1G), RIPA (strong) lysis solution (Kangwei Century Biotechnology, CW2333S), BCA protein quantification kit (Kangwei Century Biotechnology, CW0014S), skimmed milk powder (Shanghai Biyuntian Biotechnology, P0216-300g), recombinant anti-ATG7 antibody (ab133528-10), recombinant anti-ATG16L1 antibody (ab187671-10), recombinant anti-LC3B antibody (ab192890-10), recombinant anti-SQSTM1/P62 antibody (ab109012-10), recombinant anti-Beclin1 antibody (ab210498-10), recombinant anti-Bcl-xl antibody (ab32370-10), recombinant anti-P53 antibody (ab246550-10), recombinant anti-PD-L1 antibody (ab213480-10), recombinant anti-F4/80 antibody (30325T, CST), anti-GAPDH polyclonal antibody (K106389P, Solarbio), recombinant anti-Bcl-2 antibody (ab182858-10), anti-β-actin (Beijing Solarbio Technology, K101527P-100), and goat anti-rabbit IgG-HRP (Beijing Solarbio Technology, SE134-0.1).

## Method

### LLC-luc Cell and A549 Culture

LLC-luc cells are mouse LLC cells labeled with luciferase, culturing with DMEM containing 10% fetal bovine serum and 1% penicillin and streptomycin and were maintained in a 5% CO2 incubator at 37°C. Passaging in 100 mm diameter dishes once every 3 days. A549 cells are human lung cancer cells and are cultured with 1,640, while the other conditions are the same as for LLC-luc cells.

### Orthotopic Model

Taking LLC-luc cells in logarithmic growth phase, then digested the cells with 0.25% trypsin, washed and resuspended the cells with PBS, adjusted to 2×10^7^ cells/ml cell suspension, and added matrigel pre-chilled on ice at a volume ratio of 1:1, gently blow evenly and then place on ice for later use. After intraperitoneal injection of 2% pentobarbital sodium solution with 0.3 ml per 100g bodyweight of every mouse to anesthetize the mice. Lying them on the right side, the left armpit of the mice was shaved and disinfected with 75% ethanol along the left anterior axillary line for 1.5 cm up from the upper edge of the left costal arch to make a 5 mm incision (Wang et al., 2018), separating the skin and subcutaneous tissue, and the chest wall was exposed. When the pink lung lobe could be seen, a needle was vertically inserted into the left lung at a 4 mm depth, and the cell suspension was slowly injected into the left lung. Then, stopped the needle for 20 s to solidify the matrigel and slowly pulled out the needle with another hand pressing the inserted needle with a cotton swab to prevent the cell suspension from leaking. Then, sutured the incision with 1–2 stitches after removing the needle.

### Drug Preparation and Administration

The composition of YFSJ, a typical hospital preparation, is shown in Table 1 and its production process was standardized and documented in a patent (NO.201710010908.4). Briefly, primary botanical drugs of *Ganoderma lucidum*, *Cremastra appendiculata* (*D.Don*) *Makino* [Orchidaceae; *Cremastrae pseudobulbus*, *Pleiones pseudobulbus*], *Ranunculus ternatus Thunb.* [*Ranunculaceae*; *Ranunculi ternati radix*], and *Sarcandra glabra* (*Thunb.*) *Nakai* [*Chloranthaceae*; *Sarcandrae herba*] with the mass ratio of 10:5:3:4 were decocted three times. Then decoction was filtered and evaporated to obtain a thick paste before being mixed thoroughly with purified water and dextrin as well as the powder of the remaining 4 drugs with the mass ratio of 10:10:9:6. Then the mixture was heated to refine into a thick paste and rubbed onto wet pills before being spheronized and dried (more details are shown in Supplementary Table S2).

**TABLE 1:** The composition of YFSJ.

Botanical plant name	Chinese name	Chinese abbreviation
*Panax quinquefolius L.* [*Araliaceae*; *Panacis quinquefolii radix*]	Xi Yang Shen	XYS
*Fritillaria thunbergii Miq.* [*Liliaceae*; *Fritillariae thunbergii bulbus*]	Zhe Bei Mu	ZBM
*Ranunculus ternatus Thunb.* [*Ranunculaceae*; *Ranunculi ternati radix*]	Mao Zhua Cao	MZC
*Sarcandra glabra* (*Thunb.*) *Nakai* [*Chloranthaceae*; *Sarcandrae herba*]	Zhong Jie Feng	ZJF
*Cremastra appendiculata* (*D.Don*) *Makino* [Orchidaceae; *Cremastrae pseudobulbus*, *Pleiones pseudobulbus*]	Shan Ci Gu	SCG
*Pinellia ternata* (*Thunb.*) *Makino* [*Araceae*; *Pinelliae rhizoma praeparatum*]	Fa Ban Xia	BX
*Bombyx batryticatus*	Jiang Can	JC
*Ganoderma lucidum*	Ling Zhi	LZ

Mice were randomly divided into 4 groups. YFSJ (approval number of Guangdong province: Z20190015000, product batch number: 20200801) granules are formulated into 312.5 mg/ml solution at 2 times the equivalent human dose with 0.9% sterile saline. After shaking overnight at 37°C, the YFSJ solution is stored in a refrigerator at 4°C for use. Heat in a 37°C water bath before gavage and 0.8 ml YFSJ solution was administrated once for a mouse every day with a No. 8 gavage needle (YFSJ group). The autophagy inhibitor 3-methyladenine (3-MA, Selleck, S2267) and 0.9% sterile normal saline were formulated to a concentration of 2 mg/ml solution and refrigerated at 4°C. Intraperitoneal injection of 0.2 ml/time, 1 time/day (3-MA group). The tumor-bearing Model group (Model group) and the blank Control group (Control group) were given 0.8 ml of normal saline orally once every day for each mouse. YFSJ was prepared in a 2 mg/ml solution with PBS for A549 cell culture.

This mouse experiment was performed according to the protocol approved by the Animal Use and Management Institutional Committee of the Chinese Academy of Chinese Medical Sciences.

### Collection of Chemical Constituents of Botanical Drugs Contained in Yi-Fei-San-Jie-pill From the Existing Database

The chemical constituents of each botanical drug contained in YFSJ were collected from the traditional Chinese medicine systems pharmacology database (http://tcmspw.com/tcmspsearch.php, TCMSP, version 2.3) (Zhang et al., 2019). The screening condition was oral bioavailability (OB) ≥30% and drug-likeness (DL) ≥0.18. The ingredients of Jiang Can, which cannot be searched out in TCMSP, were downloaded from HIT 2.0 (http://hit2.badd-cao.net/) (Ye et al., 2011).

### Collection of Known NSCLC-Related Genes

NSCLC-related genes were obtained from Online Mendelian Inheritance in Man (OMIM, https://www.omim.org/) (Amberger et al., 2019), DrugBank database (http://www.drugbank.ca/, version 3) (Wishart et al., 2018), the Kyoto Encyclopedia of Genes and Genomes (KEGG, http://www.genome.jp/kegg/, last updated: 1 October 2021) (Kanehisa and Goto, 2000), DisGeNET (http://www.disgenet.org) (Piñero et al., 2020), GeneCards (https://www.genecards.org/, version 5.7), NCBI (https://www.ncbi.nlm.nih.gov/), PharmGKB (https://www.pharmgkb.org/) (Whirl-Carrillo et al., 2021), and Therapeutic Target Database (TTD, http://db.idrblab.net/ttd/) (Zhou et al., 2021).

### Construction of the PPI Network and Extraction of the Subnetwork

The PPI network was constructed on the basis of the public database STRING (Search Tool for Known and Predicted Protein–Protein Interactions, version 11.5, https://cn.string-db.org/) (Szklarczyk et al., 2019), with network display options hiding disconnected nodes in the network and the combined score higher than the highest confidence (0.900) of all of the combined scores. Then the interaction networks were visualized by Cytoscape software (https://cytoscape.org/, version 3.8.0) (Shannon et al., 2003). A node was defined as a gene in the network, and an edge was defined as the interaction between two genes. Hub genes were defined as nodes of topological importance and extracted via the Cytohubba (Chin et al., 2014) plug-in by the MCC algorithm (Chin et al., 2014) for further investigation.

### Enrichment Analysis of Gene Ontology and the Kyoto Encyclopedia of Genes and Genomes

Enrichment analyses of Gene Ontology (GO) and Kyoto Encyclopedia of Genes and Genomes (KEGG) pathway were performed based on the Database for Annotation, Visualization, and Integrated Discovery (Metascape, https://metascape.org/gp/index.html#/main/step1, database last update date: 2022-01-01) (Zhou et al., 2019). Only GO (Biological Processes, BP), GO (Cellular Components, CC), GO (Molecular Functions, MF) and KEGG pathways with *p*-values less than 0.01 (corrected using the Bonferroni method (Armstrong et al., 2014)) were selected and visualized by the tool “Barplot Gradient” in Hiplot (https://hiplot.com.cn) (Li et al., 2022).

### Live Imaging of Mouse Tumor

According to the results of preliminary experiments, lung tumors can be detected with the small animal live imaging instrument (IVIS lumina Series III) on the 4th day after injecting. Intraperitoneally injecting the luciferase substrate solution of 15 mg/ml with PBS for 150 ul/mouse, then putting the mice into the isoflurane anesthesia box quickly and taking images in a supine position in the small animal live imaging instrument with an exposure time of 10 s, and saving the pictures to calculate the fluorescence value.

### Western Blot

After the mice were sacrificed, the tumors were quickly stripped and collected, and 100 mg of tumor tissue was cut out (the same weight as normal lung tissue from the control group). A RIPA (strong) lysis solution containing 1% protease inhibitor was added to grind and lysate for 30 min, then centrifuged at 12,000 r·min^−1^, took the supernatant and quantified it with the BCA kit, and performed electrophoresis, transferring membrane, and blocking. After preparing the primary antibody with 5% skim milk, it was incubated overnight. Then washed the membrane with TBST, incubated the secondary antibody for 1 h, washed the membrane, and performed exposure.

### Transmission Electron Microscopy

A549 cells were pretreated with glutaraldehyde for observation of mitochondrial morphology. Cells were cut up and fixed with glutaraldehyde (4%) and then with 1% osmium tetroxide. Then the cells were dehydrated in graded ethanol and embedded in Epon. Then cell sections were preconditioned by special TEM staff before TEM observation (Hitachi H7500, Tokyo, Japan).

### Query of the Correlation of Hub Genes With Immune Infiltration, Together With Survival Outcome

The investigation/inquiry of the 15 hub genes’ roles within the immunological process was performed in the TISMO database (Zeng et al., 2021) and the correlation of hub genes with immune infiltration, together with survival outcome in lung adenocarcinoma (LUAD) and lung squamous cell carcinoma (LUSC) from TCGA, was investigated in TIMER (Li et al., 2017) and GEPIA2 (Tang et al., 2019) databases.

### Statistical Analysis

GraphPad Prism 9.0.0 (GraphPad Software, CA, United States) was used to perform a one-way analysis of variance and survival curve test in the experimental results of this study. The statistical results were expressed as X ± S. The statistical differences between groups were analyzed by one-way analysis of variance and *t* test. The difference of *p* < 0.05 is statistically different, and *p* < 0.01 indicates that the difference is markedly different.

## Results

### Yi-Fei-San-Jie-pill Inhibited Tumor Growth and Prolonged Survival Time in Orthotopic Lung Cancer Model of Mice

**FIGURE 2:**
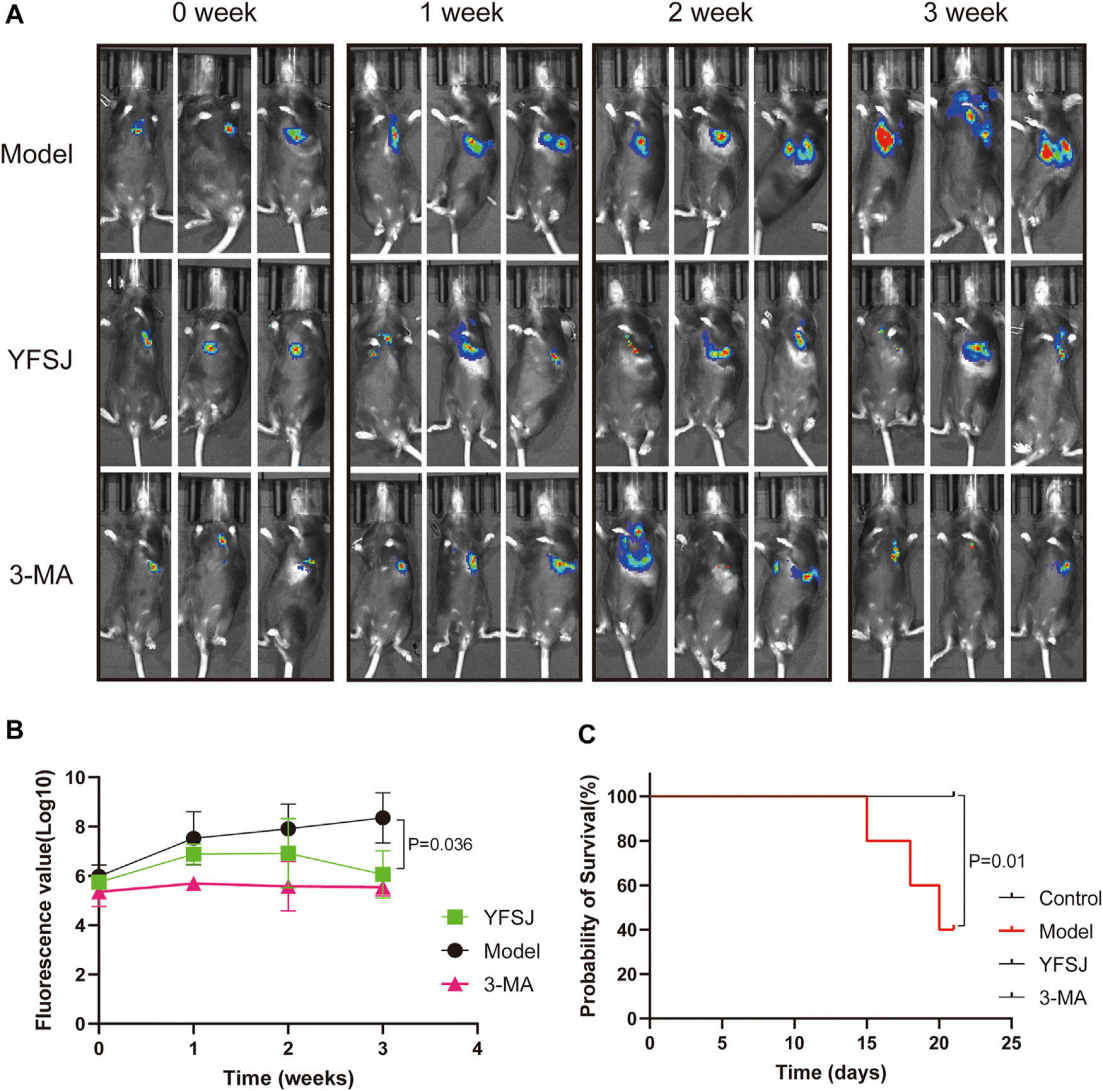
Fluorescence photography of mice in each group at 0, 1, 2, and 3 weeks of administration **(A)**. Tumor fluorescence value in each group **(B)**; survival time of mice in each group (*n* = 5) **(C)**. Model: Model group; YFSJ: Yi-Fei-San-Jie pill group; 3-MA: autophagy inhibitor 3-MA group.

As shown in Figure 2A, small animal live imaging was performed on mice at different time points, including before drug administration and 1, 2, and 3 weeks after administration. The fluorescence of lung cancer in the Model group increased dramatically, and the enhancing trend was the most significant; while, the fluorescence values of the YFSJ group and 3-MA group were ambiguous with time, but the enhancing trends were remarkably lower than that of the Model group, and YFSJ group exhibited reduction with a statistical difference (*p* < 0.05) after 3 weeks of administration. Additionally, the enhancing trend of the 3-MA group seems more stable than that of the YFSJ (Figure 2B). Figure 2C suggests that YFSJ prolonged the survival time of tumor-bearing mice, sharing the same effect as 3-MA. Nevertheless, 3 mice in the Model group died successively within 3 weeks, but there was no death in the YFSJ and 3-MA groups (*p* < 0.05). Taken together, it shows that YFSJ inhibited tumor growth and prolonged survival time in the orthotopic lung cancer Model.

### The Collection of All NSCLC Related Genes

**FIGURE 3:**
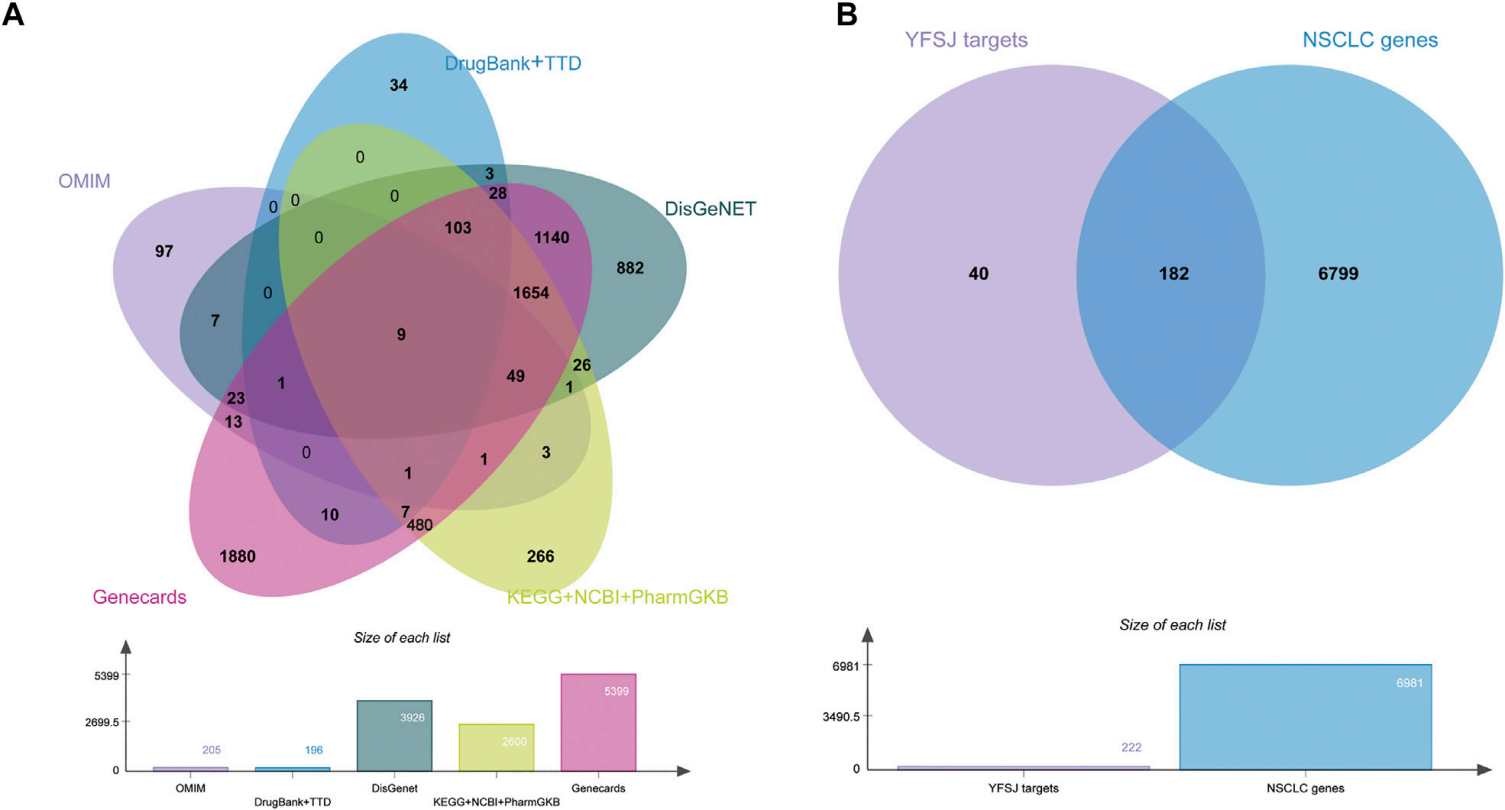
Eight databases were searched for NSCLC-related genes **(A)**. The intersection of YFSJ targets and NSCLC-related genes **(B)**.

All the NSCLC related genes were collected from as many comprehensive databases as possible and the intersection between chemical constituent targets of YFSJ and NSCLC-related genes was displayed by the Venn diagram (Chen et al., 2021). As shown in Figures 3A, 8 databases were searched for NSCLC-related genes, and a total of 6,981 genes (provided in Supplementary Data Table S1) were fully collected. YFSJ targets share 182 genes with NSCLC-related genes (Figure 3B), which accounts for a considerable extent of YFSJ targets.

### The Construction of Chemical Constituent Targets of Yi-Fei-San-Jie-pill and NSCLC-Related Genes Network

**FIGURE 4:**
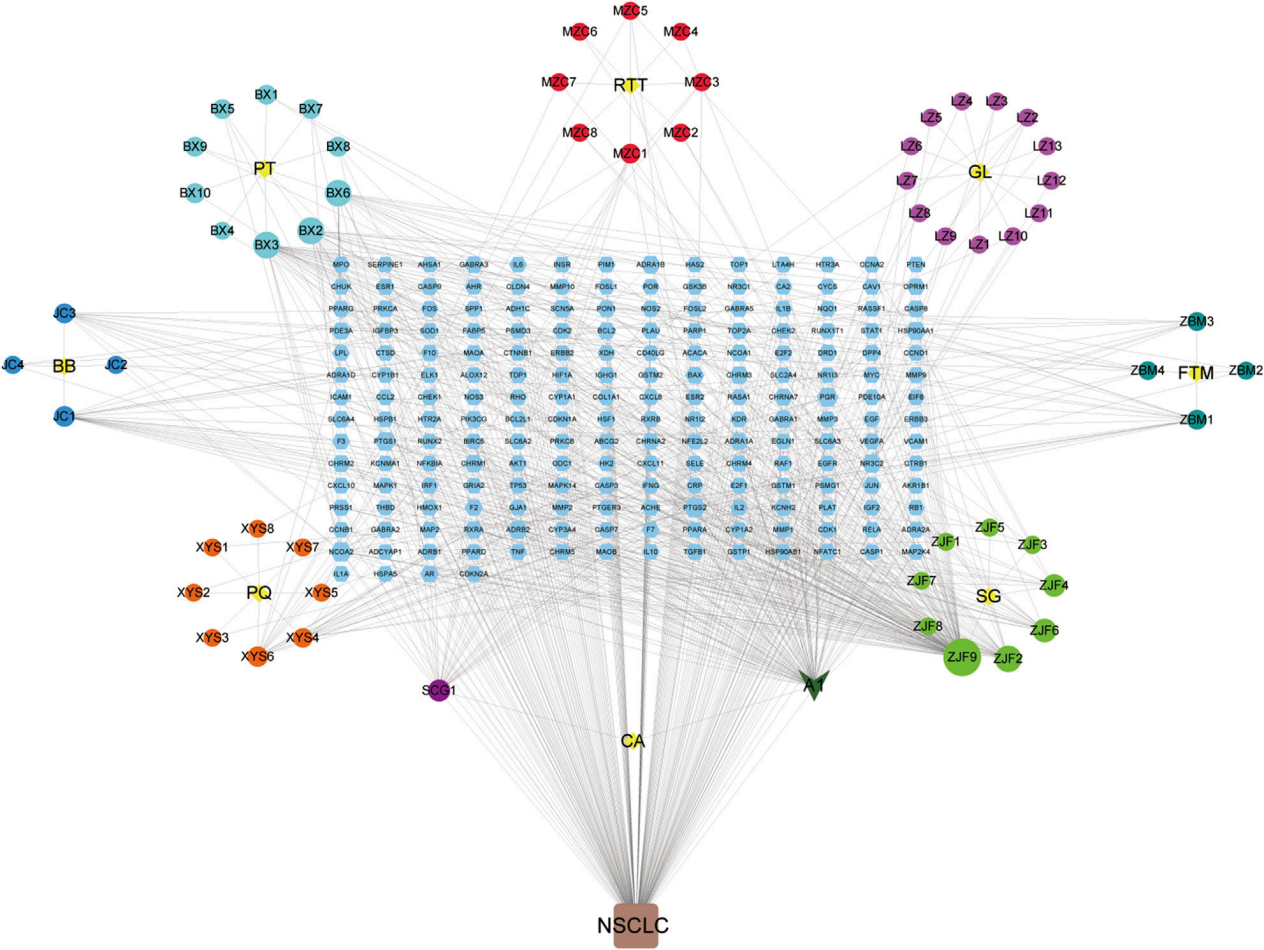
Chemical constituent targets of YFSJ and NSCLC-related genes network. The yellow nodes are eight botanical drugs of YFSJ, and the circular nodes are constituents of each drug. A1 was a shared constituent among the three (*Pinellia ternata* (*Thunb.*) *Makino* [*Araceae*; *Pinelliae rhizoma praeparatum*], *Ranunculus ternatus Thunb.* [*Ranunculaceae*; *Ranunculi ternati radix*], and *Cremastra appendiculata* (*D.Don*) *Makino* [Orchidaceae; *Cremastrae pseudobulbus*, *Pleiones pseudobulbus*] botanical drugs. The rectangular nodes are targets of chemical constituents.(PT: *Pinellia ternata* (*Thunb.*) *Makino* [*Araceae*; *Pinelliae rhizoma praeparatum*]; RTT: *Ranunculus ternatus Thunb.* [*Ranunculaceae*; *Ranunculi ternati radix*]; GL: *Ganoderma lucidum*; FTM: *Fritillaria thunbergii Miq.* [*Liliaceae*; *Fritillariae thunbergii bulbus*]; SG: *Sarcandra glabra* (*Thunb.*) *Nakai* [*Chloranthaceae*; *Sarcandrae herba*]; CA: *Cremastra appendiculata* (*D.Don*) *Makino* [Orchidaceae; *Cremastrae pseudobulbus*, *Pleiones pseudobulbus*]; PQ: *Panax quinquefolius L.* [*Araliaceae*; *Panacis quinquefolii radix*]; BB: *Bombyx Batryticatus*.).

As shown in Figure 4, YFSJ contains 8 botanical drugs (the central yellow nodes of 8 circles, provided in Supplementary Table S2), and 69 chemical constituents collected in total were represented by the circular nodes (see their standard names in Supplementary Table S3). The rectangle nodes signal 182 target genes (the symbol lists are displayed in Supplementary Table S4). A1 represented stigmasterol and was shared within *Pinellia ternata* (*Thunb.*) *Makino* [*Araceae*; *Pinelliae rhizoma praeparatum*], *Ranunculus ternatus Thunb.* [*Ranunculaceae*; *ranunculi ternati radix*], *and*

*Cremastra appendiculata* (*D.Don*) *Makino* [Orchidaceae; *cremastrae pseudobulbus*, *pleiones pseudobulbus*]. Additionally, quercetin (ZJF9) and anhydroicaritin (ZJF2) in *Sarcandra glabra* (*Thunb.*) *Nakai* [*Chloranthaceae*; *Sarcandrae herba*], cavidine (BX2), baicalein (BX3) and coniferin (BX6) in *Pinellia ternata* (*Thunb.*) *Makino* [*Araceae*; *Pinelliae rhizoma praeparatum*], and 2-methoxy-9,10-dihydrophenanthrene-4,5-diol (SCG1) of *Cremastra appendiculata* (*D.Don*) *Makino* [Orchidaceae; *Cremastrae pseudobulbus*, *Pleiones pseudobulbus*] were also accounted for critical proportion.

### PPI Network Construction and Hub Genes Extraction

The 182 intersectional genes were searched in STRING for PPI network construction. As shown in Supplementary Figure S1A, the PPI network contains 181 nodes and 728 edges. The average node degree is 8.04, and the average local clustering coefficient is 0.419; the PPI enrichment *p*-value being less than 1.0e-16. The Cytohubba plug-in was employed to extract hub genes via the MCC algorithm. The hub genes subnetwork (Supplementary Figure S1B) contains 15 top genes (Table 2): interleukin 1 beta (*IL1B*), C-X-C motif chemokine ligand 8 (*CXCL8*), interleukin *6* (*IL6*), tumor necrosis factor (*TNF*), interleukin 1 alpha (*IL1A*), BHLH transcription factor (*MYC*), NF-KB subunit (*RELA*), jun proto-oncogene, AP-1 transcription factor subunit (*JUN*), C-C motif chemokine ligand 2 (*CCL2*), hypoxia-inducible factor 1 subunit alpha (*HIF1A*), mitogen-activated protein kinase 1 (*MAPK1*), fos proto-oncogene, AP-1 transcription factor subunit (*FOS*), cyclin D1 (*CCND1*), tumor protein p53 (*TP53*), and estrogen receptor 1 (*ESR1*)*.* It is speculated that the 15 top genes may play pivotal roles in ameliorating the lung tumor effect of YFSJ.

**TABLE 2:** Top 15 genes extracted from PPI ranked by the MCC algorithm.

Rank	Name	Score
1	*JUN*	19,600
2	*RELA*	18,582
3	*TNF*	12,622
4	*MYC*	12,246
5	*IL6*	11,772
6	*ESR1*	11,412
7	*IL1A*	10,808
8	*CCL2*	10,800
9	*IL1B*	10,360
10	*CXCL8*	10,206
11	*FOS*	8,806
12	*HIF1A*	8,768
13	*CCND1*	7,786
14	*TP53*	7,103
15	*MAPK1*	6,177

The meaning of the italic values represents the scores that were calculated using the MCC algorithm to evaluate the relationship between nodes and edges. The higher the score, the more important the gene is.

### Gene Ontology and the Kyoto Encyclopedia of Genes and Genomes Enrichment of Yi-Fei-San-Jie-pill’s Inhibitive Effect on Lung Tumor

**FIGURE 5:**
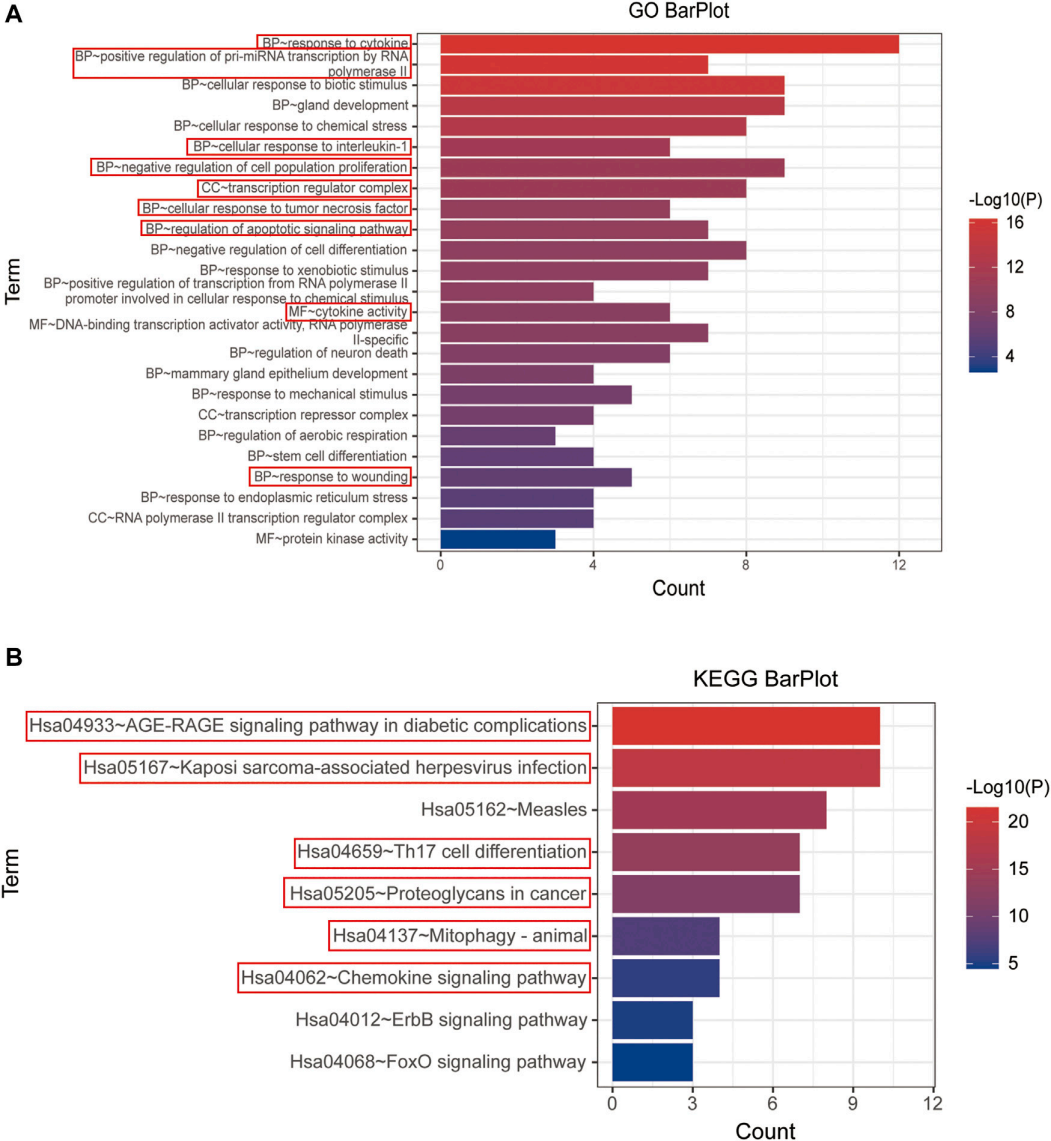
GO enrichment of 15 top genes extracted from PPI network **(A)**. BP: biological process, CC: cell component, and MF: molecular function. KEGG enrichment of 15 top genes extracted from PPI network **(B)**. The items marked with red boxes represent the very important GO processes and KEGG pathways that directed to further analysis.

Both GO and KEGG enrichment of 15 hub genes were performed in the Metascape database for details of YFSJ’s inhibitive effect on lung tumor. As exhibited in Figure 5A, the 15 top genes were enriched mainly in BP of response to cytokines, positive regulation of pre-miRNA transcription by RNA polymerase II, cellular response to interleukin-1, negative regulation of cell population proliferation, cellular response to tumor necrosis factor, regulation of apoptotic signaling pathway, and response to wounding which were highlighted by red boxes and the same went for the following. While, CC of the transcription regulator complex and MF of cytokine activity were primarily enriched. Also, the KEGG enrichment (Figure 5B) suggests that the 15 top genes mainly affect the AGE-RAGE signaling pathway in diabetic complications (sub-pathways like TNF signaling pathway, MAPK signaling pathway, NF-κB signaling pathway, and necroptosis), Kaposi sarcoma-associated herpesvirus infection (sub-pathways like pathways in cancer and cellular senescence), Th17 cell differentiation (sub-pathways like apoptosis, PD-L1 expression and PD-1 checkpoint pathway in cancer, B-cell receptor signaling pathway, HIF-1 signaling pathway, and estrogen signaling pathway), proteoglycans in cancer, mitophagy, and chemokine signaling pathway. Taken together, the enrichments mentioned earlier imply that fundamental biological processes such as apoptosis, au (mi) tophagy, cancer-related pathway, cytokine/immune-related pathway, and miRNA biological process might be involved in the anti-tumor impact of YFSJ.

### Yi-Fei-San-Jie-pill Was Verified to Inhibit the Expression of Apoptosis-Related Proteins and Autophagic Proteins and to Interfere With Mitophagy

**FIGURE 6:**
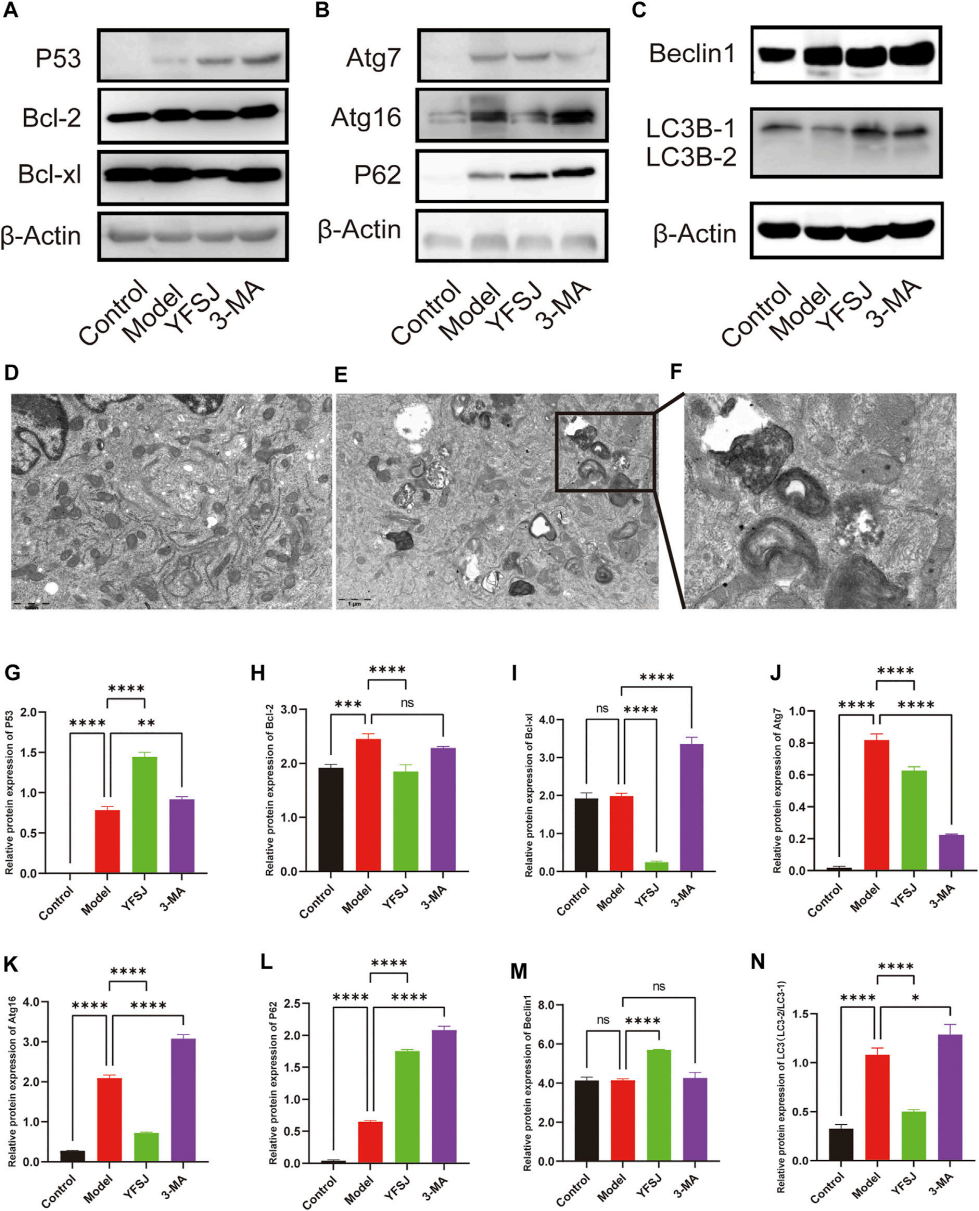
Western blot of apoptosis-related and autophagy-related proteins **(A,B,C,G-N)**. Transmission electron microscope (20,000×) of Control **(D)** and YFSJ **(E,F)** treated A549 cell.

The anti-apoptosis proteins Bcl-2 and Bcl-xl in the Model group were increased compared with those in the control group (Figures 6A,H,I), especially Bcl-2 which significantly increased (Figure 6H), indicating that the tumor cells gained anti-apoptotic ability remarkably. YFSJ significantly reduced the expression of Bcl-2 (Figure 6H) and Bcl-xl proteins (Figure 6I). Meanwhile, compared with the Model group, 3-MA reduced the expression of Bcl-2 in tumor-bearing mice, but there was no statistical difference (Figure 6H); while 3-MA remarkably increased the expression of Bcl-xl (Figure 6I), which was the same as that of the YFSJ group. The effect of Bcl-xl protein expression was the opposite, suggesting that 3-MA had no obvious effect on the apoptosis mechanism. P53, the well-known anti-apoptosis protein, was significantly upregulated in the YFSJ and 3-MA groups (Figures 6A,G). In addition, autophagy proteins in the Model group were generally upregulated compared with those in the Control group, suggesting that autophagy in tumor cells was generally enhanced. YFSJ remarkably reduced Atg16 (Figures 6B,K), Atg7 (Figures 6B,J) and LC3-2/LC3-1 ratio (Figures 6C,N), significantly increasing the expression of P62 (Figures 6B,L), suggesting that YFSJ remarkably inhibited tumor autophagic activities and subsequently blocked autophagy flow, which was shown by the accumulation of P62. 3-MA also significantly inhibited the expression of Atg7 (Figures 6B,J) and increased the expression of P62 (Figures 6B,L), which was more obvious than that of the YFSJ group, but Atg16 (Figures 6B,K) and LC3-2/LC3-1 ratio (Figures 6C,N) were remarkably higher than that in the Model group, suggesting that 3-MA may mainly target to Atg7 and its downstream pathways specifically to interfere with autophagy. Both 3-MA and YFSJ did not significantly inhibit Beclin1 protein expression (Figures 6C,M), which, however, was even upregulated by YFSJ, suggesting that the regulation of autophagy by both 3-MA and YFSJ is Beclin1-independent in the mouse orthotopic lung cancer Model of this study. According to the mitophagy pathway of the enrichment from 15 top genes, we detected the A549 cell transmission electron microscope after YFSJ administration. Figure 6D is the Control group, and Figures 6E,F are the YFSJ group, which shows that YFSJ interfered with mitophagy.

### Immune Search of Preclinical Lung Cancer Model Together With the Correlation of Hub Genes With Immune Infiltration

**FIGURE 7:**
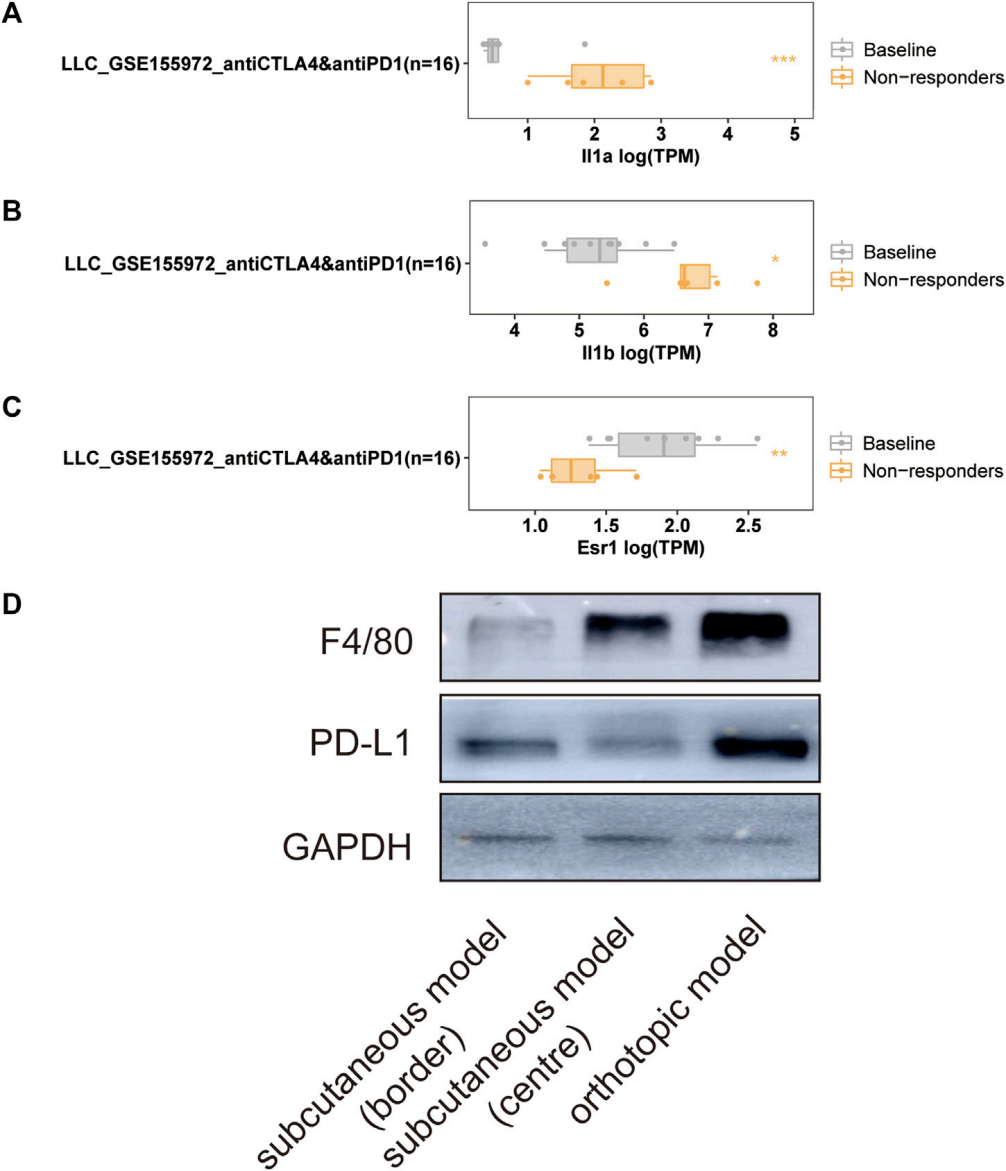
Significantly different expressions of *IL1A* **(A)**, *IL1B* **(B)**, and *ESR1* **(C)** from the LLC mouse model in different groups of mice treated with immune checkpoint inhibitors. The markers of macrophage (F4/80) and classic immune checkpoint (PD-L1) inhibitor were expressed differently among the border, the center of the subcutaneous model, and the orthotopic model of LLC **(D)**.

**FIGURE 8:**
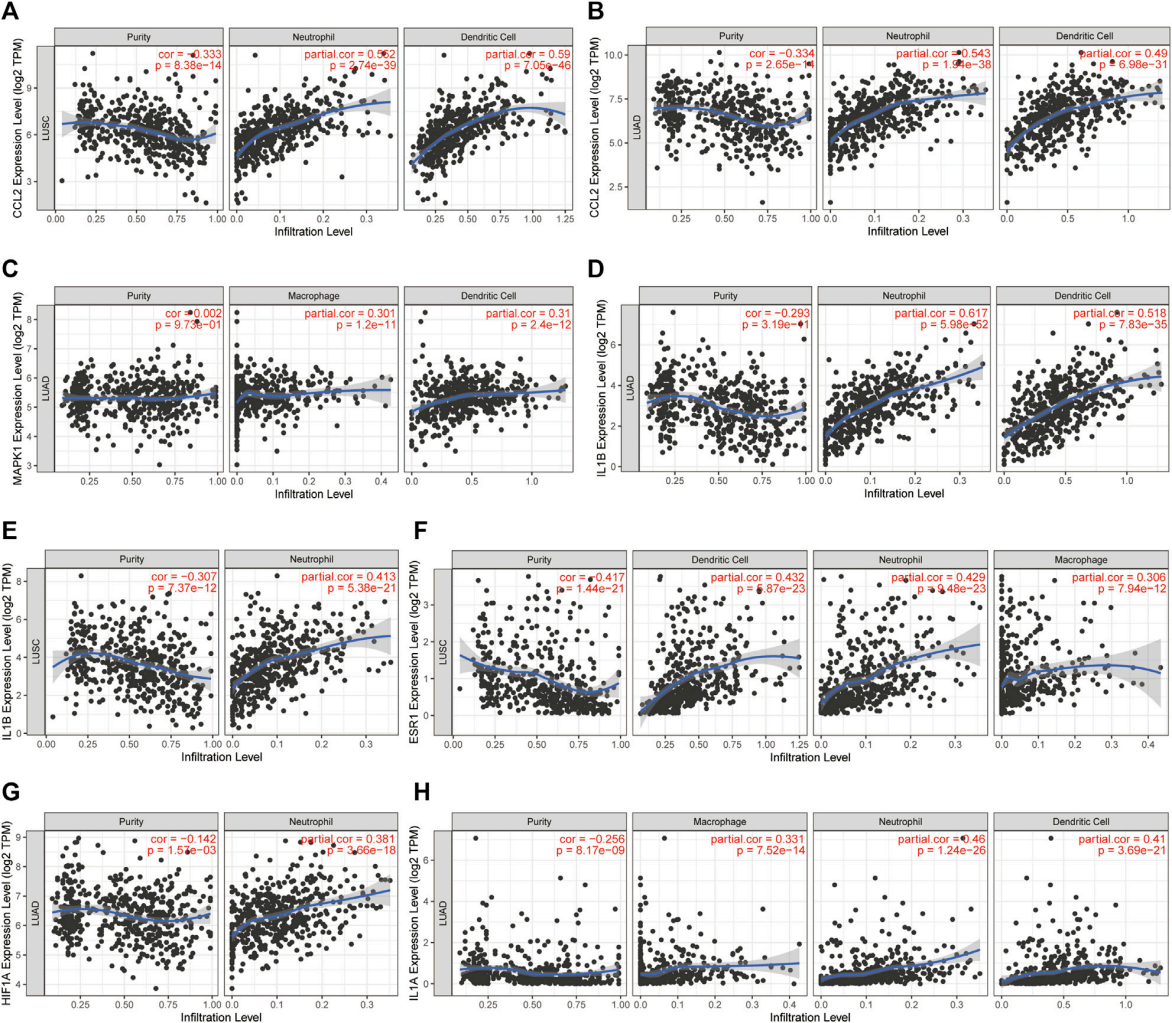
The correlation between *CCL2* **(A,B)**, *MAPK1* **(C)**, *IL1B* **(D,E)**, *ESR1* **(F)**, *HIF1A* **(G)**, and *IL1A* **(H)** from 15 hub genes with different immune cells infiltrated in lung adenocarcinoma (LUAD) and/or lung squamous cell carcinoma (LUSC) from TCGA.

The LLC mouse Model was widely applied in cancer research, and the TISMO database was established (Zeng et al., 2021) because of the oncology immunotherapy burst. The enrichment of 15 hub genes implied that YFSJ might inhibit orthotopic lung tumors via immune-related targets or pathways. Further inquiry of the 15 hub genes’ roles within the immunological process was performed in the TISMO database and *IL1A* (Figure 7A) and *IL1B* (Figure 7B) were expressed higher in non-responders than in baseline in LLC mice Model treated with anti-CTLA4 and anti-PD1, which indicated that *IL1A* and *IL1B* may be the factors that blunted the effect of immune checkpoint inhibitors (ICI). While, *ESR1* displayed the opposite trend (Figure 7C) and implied a positive role in ICI treatment. It was obvious that *IL1A*, *IL1B*, and *ESR1* may have played important roles in the immunotherapy of the LLC mice Model. To date, most pre-clinical mice models have been subcutaneous, which, however, inevitably differ from orthotopic models, especially in the immunological process. Hence, we detected the markers of macrophage (F4/80) and classic immune checkpoint PD-L1. As shown in Figure 7D, F4/80 and PD-L1 expression were higher in the orthotopic model than in the subcutaneous model, which explicitly indicated that the immune process (e.g., innate immune) was far more fully involved in orthotopic than the subcutaneous model. Interestingly, F4/80 was expressed higher in the center than that of the border in the subcutaneous model, while, conversely, PD-L1 was lower in the center than that of the border in this model. The inquiry of hub genes was also performed in TCGA via TIMER and the correlation between the genes of *CCL2* (Figures 8A,B), *MAPK1* (Figure 8C), *IL1B* (Figures 8D,E), *ESR1* (Figure 8F), *HIF1A* (Figure 8G), *IL1A* (Figure 8H), and different immune cells (e.g., neutrophil, dendritic cell, and macrophage that belong to innate immune) infiltrated in lung adenocarcinoma (LUAD) and/or lung squamous cell carcinoma (LUSC) was positively significant. Whereas, the correlation of genes and purity of infiltration level displayed negative significant. Taken together, these outcomes hinted that it might be innate immune that accounted for immunological process involved in YFSJ targeting lung cancer.

### Immune Related Survival of Hub Genes Investigation

**FIGURE 9:**
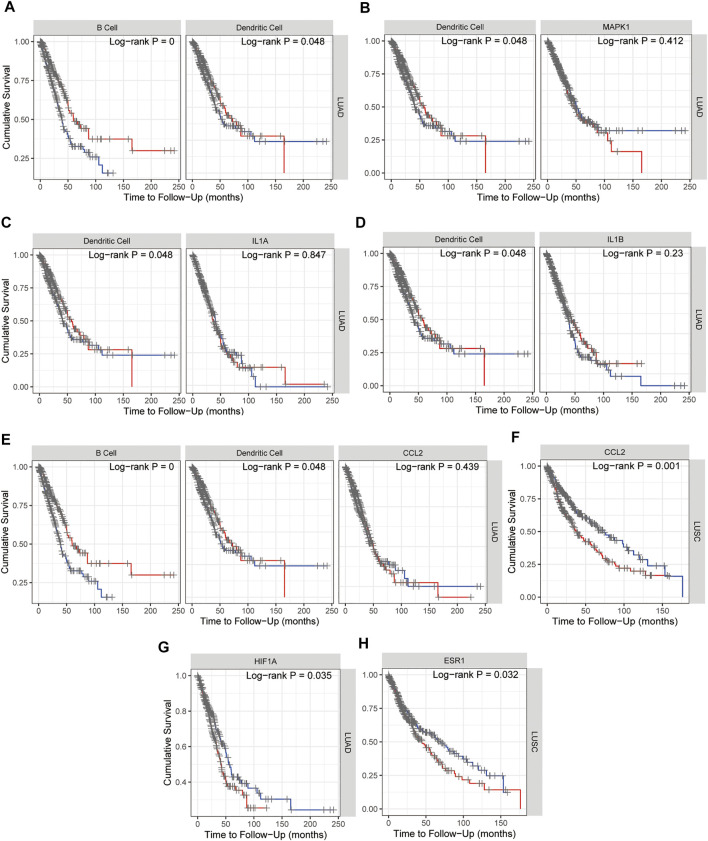
Kaplan–Meier plots for immune infiltrates of lung adenocarcinoma (LUAD) and/or lung squamous cell carcinoma (LUSC) from TCGA **(A)** and *MAPK1* **(B)**, *IL1A* **(C)**, *IL1B* **(D)**, *CCL2* **(E,F)**, *HIF1A* **(G)**, and *ESR1* **(H)** from 15 hub genes to visualize the survival differences. Levels are divided into low and high levels by 50% and the red curve represents the high top 50% while the blue curve represents the low bottom 50%. The *p*-value of the log-rank test for comparing survival curves of two groups is shown in each plot, and *p* < 0.05 was considered significantly different.

Kaplan–Meier plots for immune infiltrates of lung adenocarcinoma (LUAD) and/or lung squamous cell carcinoma (LUSC) from TCGA and *MAPK1*, *IL1A*, *IL1B*, *CCL2*, *HIF1A*, and *ESR1* from 15 hub genes were also performed in TIMER and as shown in Figure 9, B cell and Dendritic cell (DC) in LUAD (Figure 9A) and *MAPK1* in LUAD (Figure 9B), *IL1A* (Figure 9C) and *IL1B* (Figure 9D) with DC, *HIF1A* (Figure 9G) and *CCL2* with B cell and DC in LUAD (Figure 9E), *CCL2* (Figure 9F), and *ESR1* (Figure 9H) in LUSC indicated the survival differences, which hinted that *MAPK1*, *IL1A*, *IL1B*, *CCL2*, *HIF1A*, and *ESR1* might potentially serve as prognostic signatures together with B cell or dendritic cell. The survival map of 15 hub genes in lung adenocarcinoma (LUAD) and lung squamous cell carcinoma (LUSC) from TCGA was investigated in GEPIA2, and as Supplementary Figure S2 showed that in LUSC, *ESR1* and *CCL2* are significantly positive in the hazard ratio (HR). While in LUAD, *CCND1*, *MAPK1*, *HIF1A*, and *RELA* are significantly positive in HR. Taken together, the survival map confirmed that *ESR1*, *CCL2*, *MAPK1*, and *HIF1A* may predict a poor prognosis of NSCLC.

## Discussion

Although new therapies are emerging continuously, there is still a lack of better therapies with higher response rates and fewer side effects for NSCLC. TCM has a long history of treating lung cancer with definite effects and fewer side effects and can promote the survival of patients (Teng et al., 2020), which is worthy of in-depth illustration. Based on TCM theory, the pivotal pathogenesis of NSCLC is the weakness of Qi with phlegm and blood stasis, which plays a fundamental role throughout the oncogenic and progressive process of NSCLC (Wang J. et al., 2021). Weakness of Qi is the primary cause of tumors and blood stasis in the subsequent course. YFSJ, directed against the basis of the TCM theory of “phlegm, blood stasis, poison and deficiency” for NSCLC, consisting of *Panax quinquefolius L.* [*Araliaceae*; *Panacis quinquefolii radix*], *Ganoderma lucidum*, *Fritillaria thunbergii Miq.* [*Liliaceae*; *Fritillariae thunbergii bulbus*], *Cremastra appendiculata* (*D.Don*) *Makino* [Orchidaceae; *Cremastrae pseudobulbus*, *Pleiones pseudobulbus*], *Ranunculus ternatus Thunb.* [*Ranunculaceae*; *Ranunculi ternati radix*], *Sarcandra glabra* (*Thunb.*) *Nakai* [*Chloranthaceae*; *Sarcandrae herba*], *Bombyx batryticatus*, and *Pinellia ternata* (*Thunb.*) *Makino* [*Araceae*; *Pinelliae rhizoma praeparatum*] is the basic applicant formula for the treatment of NSCLC in the Cancer Center of the First Affiliated Hospital of Guangzhou University of Chinese Medicine. Prospective randomized controlled clinical trials were performed on nearly 300 patients with stage III and IV NSCLC from 6 hospitals across China, which revealed that the median survival time reached 477 days, maintaining a high quality of life as well as less economic burden (Zhou et al., 2014). Figure 4 displayed that the nodes connected with more edges of 69 chemical constituents may be the key chemicals in YFSJ that carry out the anti-tumor effects. For example, A1 (Stigmasterol) was the most shared component among different botanical drugs of YFSJ and almost have the most abundant edges with other nodes. Then, ZJF9/2/6, SCG1, XYS6/4, JC1/3, BX3/2/6, LZ10, ZBM1/3, and MZC1/3 also have more edges than the others. According to TCM theory, YFSJ contains 2 groups of botanical drugs: those reinforcing Qi and nourishing Yin (FuZheng) group and removing phlegm and blood stasis (QuXie) group. The former group includes XYS and LZ, and the latter, the rest of the botanical drugs of YFSJ. Therefore, we concluded that XYS6/4 and LZ10 may be the main components that carry out the “reinforcing Qi and nourishing Yin” function, and ZJF9/2/6, SCG1, JC1/3, BX3/2/6, ZBM1/3, and MZC1/3 may be the core components to remove phlegm and blood stasis. When we searched for the pharmacologic action of these components, the literature showed that XYS6/4 and LZ10 stimulated immune function (Li X. et al., 2020) and exhibited anti-proliferation, anti-invasion, anti-metastasis effects, and delayed AD progression (Lai et al., 2019), which was consistent with the design that XYS and LZ were targeted for strengthening Qi and with the pathophysiology that weakness of Qi was primary cause of NSCLC.

As to removing phlegm and blood stasis (QuXie) group, ZJF9 (quercetin) was reported to reduce Bcl-2 and enhance apoptosis in NSCLC (Alam et al., 2022), inhibit the proliferation and metastasis of NSCLC via the Fn14/NF-κB pathway (Dong et al., 2020), which was in accordance with the inhibiting Bcl-2 of YFSJ in the present study. JC1 (beta-carotene) was reported to inhibit the activation of MAPK in the ferret lung cancer model (Kim et al., 2006), and this echoes back that *MAPK1* predicts the prognosis in Figure 8D. BX3 (baicalein) was reported to induce mitochondrial fission, apoptosis and regulate autophagy in an LLC xenograft model (Deng et al., 2020), inhibit NSCLC invasion and metastasis by regulating the inflammation microenvironment (Zhang J et al., 2020), which agrees with the conclusion of the present study that apoptosis, autophagy and even the inflammation-related factors that 15 hub genes containing played a significant role in YFSJ’s inhibitive effect of NSCLC. At present, most animal lung cancer experiments generally adopt subcutaneous tumor models, which are convenient to operate and have high tumor formation rates. Whereas the subcutaneous tumor model cannot simulate the entirely natural process of tumor initiation, development, and even metastasis in the organs or can reflect the crosstalk between tumor cells and the tumor microenvironment. In addition, given the perspective of TCM, the lung cancer subcutaneous tumor model does not reflect the onset and progression of the tumor in the lung and lung meridian of TCM, and it deviates tremendously from the lung cancer patients in a real-world setting. Therefore, it is not conducive to the research of TCM based on the theory of visceral outward manifestation and meridian. It is also found that some TCM botanical drugs are effective in orthotopic transplantation of lung cancer model mice, but there is poor or no effect in subcutaneous transplantation model mice (Jacoby et al., 2010), which indicates that the orthotopic cancer model and the subcutaneous cancer model are almost totally different. It was confirmed in the present study that immune checkpoint PD-L1 and F4/80, the marker of macrophages, were detected to express significantly higher in the orthotopic model than in the subcutaneous one. The orthotopic cancer model tends to mimic the process of tumorigenesis, immunoediting, and immune escape of lung cancer in mice, providing a prerequisite for the interpretation of the TCM mechanism and tumor immunotherapy.

In addition, protein–protein interactions are mainly offered by the STRING database according to confidence score, and all protein interaction data were weighted, integrated, and therefore have a reliable calculated value (confidence score), enabling users to screen large-scale target protein networks by simply selecting the confidence score. Since the CytoHubba plug-in was common in hub genes extraction (Ma et al., 2021), we ran it via the MCC algorithm, which was reported to be superior to others in the CytoHubba. The hub genes extracted from the PPI network of YFSJ targets and NSCLC targets intersection have enriched BP (e.g., cellular response to interleukin-1, response to xenobiotic stimulus, and response to wounding) and pathways (Th17 cell differentiation) involved in innate immunity. Innate immunity executes as a fundamentally defensive system and has been exploited for novel immunotherapy of kinds of cancers (Kalafati et al., 2020; Priem et al., 2020). Further cancer immunological database search verified that the 15 hub genes are mainly related to neutrophils, dendritic cells, and macrophages, which belong to innate immunity. They have a significant correlation with the prognostic outcome at different degrees and may stand for potential mechanical elucidation and even novel drug discovery (e.g., natural product drugs) (Gupta et al., 2019; Sun et al., 2020).

Lung cancer has been reported to be closely related to apoptosis and autophagy. Apoptosis serves an important role in the process of cell life. Most cancer cells have the properties of inhibiting apoptosis program so as to gain immortality (Hanahan and Weinberg, 2011). For example, a recent study found that naproxen inhibited the formation of spontaneous lung adenocarcinoma in Kras-mutant mice by significantly increasing the apoptosis of cancer cells (Kumar et al., 2021). It was believed that promoting the apoptosis of senescent cells or cancer cells is one of the main forms of anti-cancer (Pentimalli et al., 2018). In this study, Bcl-2 and Bcl-xl, which are generally highly expressed in cancer cells (Yang et al., 2019; Tanimura et al., 2021), were detected for their anti-apoptotic properties. Bcl-xl was not remarkably upregulated in the model group compared to the control group, which may be related to the cell line used in this study. For example, in the lung cancer brain metastasis model constructed by the human NSCLC PC-9 cell line, Bcl-2 was also significantly upregulated, while Bcl-xl is not remarkably upregulated (Liu et al., 2021), suggesting that the expression of apoptosis-related proteins depends on the cancer cell lines. Autophagy is usually hijacked by cancer cells to survive the energy crisis caused by rapid proliferation and the cellular stress caused by anticancer drugs. Recent studies have shown that inhibiting autophagy enables curcumin to overcome the primary resistance of NSCLC cells to gefitinib (Chen et al., 2019), and regulating mitophagy, the autophagy of mitochondria, was reported to inhibit cancer cells (Chang et al., 2018; Li et al., 2019; Huang et al., 2020). As it is known to all that cancer cells need more energy and rely on enhanced glycolysis. Nonetheless, mitochondria were corroborated to promote aerobic oxidation for lung cancer survival and metastasis (Li F. et al., 2020; Sorrenti et al., 2021). The latest review also shows that the combination of traditional therapies and autophagy inhibitors including 3-MA can improve the efficacy of lung cancer (Wang Z. et al., 2021), and even intervention in specific autophagy genes can improve the response rate of immunotherapy (Lawson et al., 2020). We have revealed that YFSJ-treated patients express higher microRNA-182 than those in the control group, and microRNA-182 was predicted and verified to target Atg7 (data not shown). In this study, positive regulation of pre-miRNA transcription by RNA polymerase II (BP) was enriched, and YFSJ showed a significant inhibitory effect on Atg7 and Atg16, manifesting an extensively blunting autophagy effect. However, we found YFSJ significantly increased Beclin1 expression, compared to the model group, rather than decreasing its protein level, which suggests that YFSJ regulated autophagy through a Beclin1-independent pathway. Indeed, it has been periodically reported that many natural products interfere with tumor cell autophagy through Beclin1-independent pathways, such as resveratrol (Scarlatti et al., 2008), arsenic trioxide (Smith et al., 2010), and so on. Considering that the BH3 domain of Beclin1 can combine with Bcl-2 to form a complex (Lee et al., 2019), it is speculated that YFSJ may release more Beclin1 after inhibiting Bcl-2.

This study confirmed that YFSJ inhibits tumor growth and prolongs survival time by promoting tumor cell apoptosis, inhibiting tumor cell autophagy, which is consistent with the results of the previous research of our research team (Zhang X et al., 2020); thereby providing a reference for its further clinical application. Additionally, due to objective reasons, this study lacks more samples and inhibitor rescue experiments. Future studies are warranted to increase the sample volume for further verification and increase inhibitor experiments for further exploration of downstream pathways.

## Conclusion

This study provides convincing evidence that YFSJ inhibited the growth of lung cancer and prolonged the survival time of tumor-bearing mice based on the NSCLC orthotopic model, and its anti-tumor effect was closely related to the promotion of apoptosis, interfering with autophagy and immune infiltration.

## Data Availability

The raw data supporting the conclusions of this article will be made available by the authors, without undue reservation.

## Abbreviations

YFSJ, Yi-Fei-San-Jie(-pill); NSCLC, non-small cell lung cancer; 3-MA, 3-methyladenine; TCM, traditional Chinese medicine; LLC, Lewis lung cancer; TCMSP Database, traditional Chinese medicine systems pharmacology database; OMIM Database, Online Mendelian Inheritance in Man Database; KEGG Database, the Kyoto Encyclopedia of Genes and Genomes Database; TTD Database, Therapeutic Target Database; PPI, protein–protein interaction; GO, gene ontology; GO (BP), gene ontology (biological process); GO (CC), gene ontology (cell component); GO (MF), gene ontology (molecular function); TEM, transmission electron microscopy; LUAD, lung adenocarcinoma; LUSC, lung squamous cell carcinoma; TISMO, Tumor Immune Syngeneic Mouse database; TCGA, The Cancer Genome Atlas Database; ICI, immune checkpoint inhibitors; TIMER, tumor immune estimation resource database; GEPIA2, gene expression profiling interactive analysis database, Version 2; BX, Banxia (*Pinellia ternata* (*Thunb.*) *Makino* [*Araceae*; *Pinelliae rhizoma praeparatum*]); JC, Jiangcan (*Bombyx Batryticatus*); LZ, Lingzhi (*Ganoderma Lucidum*); MZC, Maozhuacao (*Ranunculus ternatus Thunb.* [*Ranunculaceae*; *Ranunculi ternati radix*]); SCG, Shancigu (*Cremastra appendiculata* (*D.Don*) *Makino* [Orchidaceae; *Cremastrae pseudobulbus*, *Pleiones pseudobulbus*]; XYS, Xiyangshen (*Panax quinquefolius L.* [*Araliaceae*; *Panacis quinquefolii radix*]); ZBM, Zhebeimu (*Fritillaria thunbergii Miq.* [*Liliaceae*; *Fritillariae thunbergii bulbus*]; ZJF, Zhongjiefeng (*Sarcandra glabra* (*Thunb.*) *Nakai* [*Chloranthaceae*; *Sarcandrae herba*]); FoxO, forkhead box O.
