# Supplementary figures and images for: Deciphering the Effects and Mechanisms of Yi-Fei-San-Jie-pill on Non-Small Cell Lung Cancer With Integrating Network Target Analysis and Experimental Validation
Source: Front Pharmacol. 2022 May 11;13:851554. doi: 10.3389/fphar.2022.851554 (PMC9130494; doi:10.3389/fphar.2022.851554)

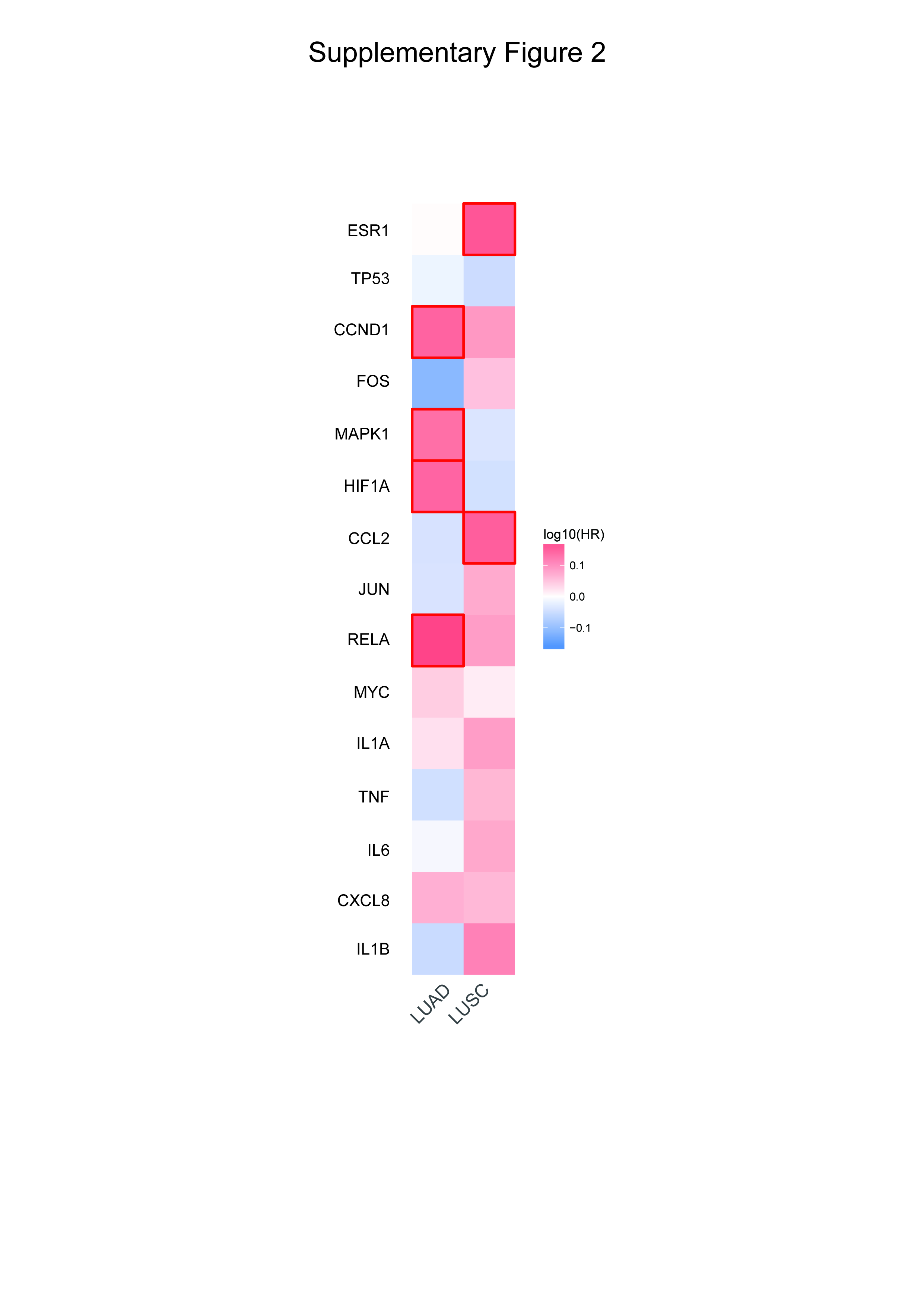

Supplement: Supplementary file 3 [file Image2.TIF]

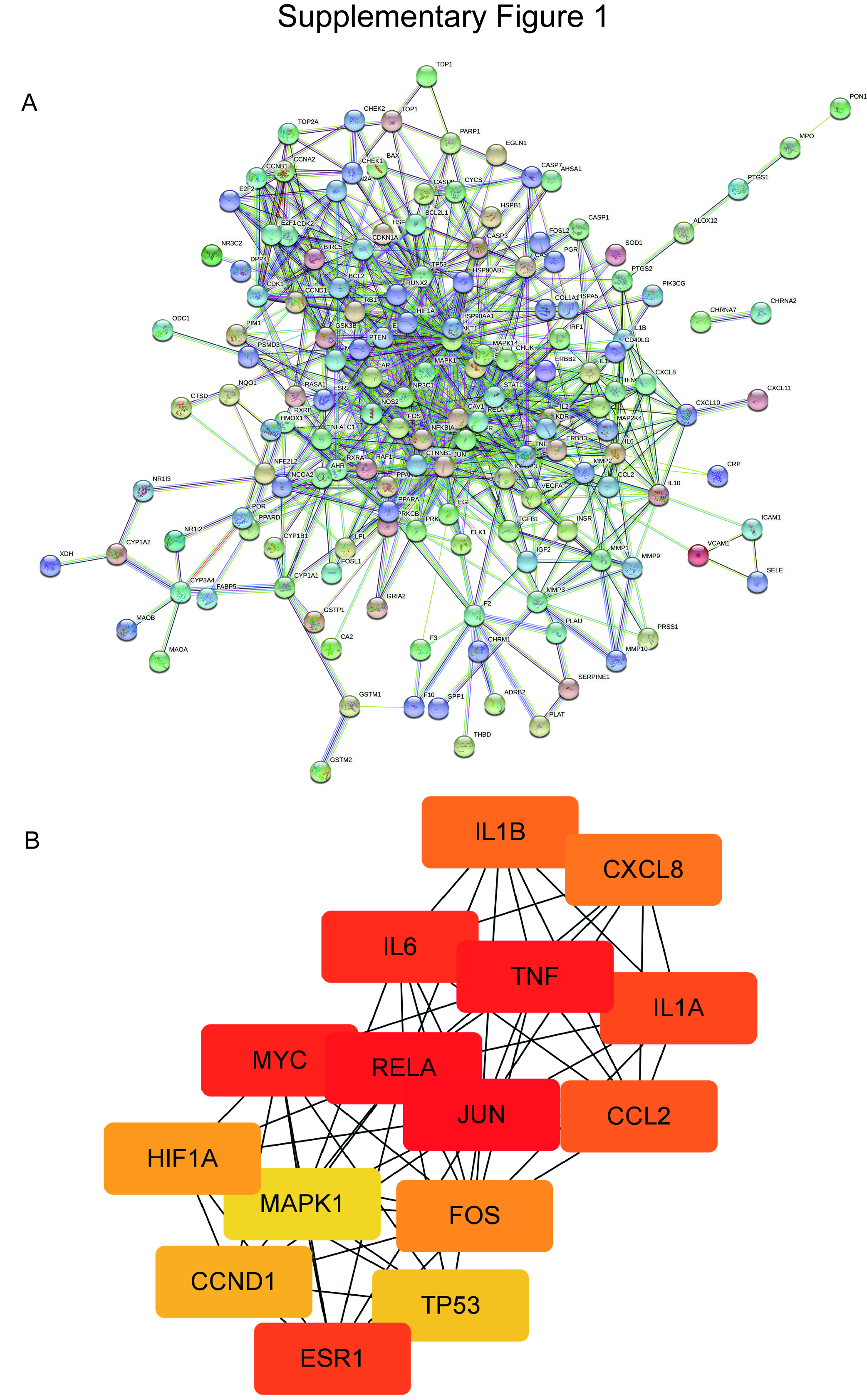

Supplement: Supplementary file 4 [file Image1.TIF]
