# Supplementary material for: Deciphering the Effects and Mechanisms of Yi-Fei-San-Jie-pill on Non-Small Cell Lung Cancer With Integrating Network Target Analysis and Experimental Validation
Source: Front Pharmacol. 2022 May 11;13:851554. doi: 10.3389/fphar.2022.851554 (PMC9130494; doi:10.3389/fphar.2022.851554)
Supplement: Supplementary file 5 [file DataSheet1.PDF]

Accepted name from MPNS V11	Chinese name	Chinese phonetic Abbreviation
Panax quinquefolius L. [Araliaceae;panacis quinquefolii radix]	Xi Yang Shen	XYS
Fritillaria thunbergii Miq. [Liliaceae;fritillariae thunbergii bulbus]	Zhe Bei Mu	ZBM
Ranunculus ternatus Thunb. [Ranunculaceae;ranunculi ternati radix]	Mao Zhua Cao	MZC
Sarcandra glabra (Thunb.) Nakai [Chloranthaceae;sarcandrae herba]	Zhong Jie Feng	ZJF
Cremastra appendiculata (D.Don) Makino [Orchidaceae;cremastrae pseudobulbus, pleiones pseudobulbus]	Shan Ci Gu	SCG
Pinellia ternata (Thunb.) Makino [Araceae;pinelliae rhizoma praeparatum]	Fa Ban Xia	BX
*bombyx batryticatus**	Jiang Can	JC
*Ganoderma lucidum**	Ling Zhi	LZ

**bombyx batryticatus* and *Ganoderma lucidum* cannot be searched out in *http://mpns.kew.org/mpns-portal/ or http://www.plantsoftheworldonline.org/ or as a last resource www.theplantlist.org*. Therefore, we found their correct names in TCMSP, HIT2 databases and literatures of *Frontiers in Pharmacology*.

The prepared and extracted procedures of Yi-Fei-San-Jie-Pill (YFSJ) were as follows:

Procedure 1:

Primary botanical drugs of *bombyx batryticatus*, *Fritillaria thunbergii Miq. [Liliaceae;fritillariae thunbergii bulbus], Pinellia ternata (Thunb.) Makino [Araceae;pinelliae rhizoma praeparatum]* and *Panax quinquefolius L. [Araliaceae;panacis quinquefolii radix]* with the mass ratio of 10:10:9:6 were ground into powder and filtered through a 100-mesh sieve, which was named extract S1.

Procedure 2:

Primary botanical drugs of *Ranunculus ternatus Thunb. [Ranunculaceae;ranunculi ternati radix], Sarcandra glabra (Thunb.) Nakai [Chloranthaceae;sarcandrae herba], Cremastra appendiculata (D.Don) Makino [Orchidaceae;cremastrae pseudobulbus, pleiones pseudobulbus]*and *Ganoderma lucidum* with the mass ratio of 10:5:3:4 were decocted three times in eight, six and four volumes of water for 1.5h, 1.0h and 0.5h, respectively. Then discarded the dross of the 4 botanical drugs and retained the decoction. Then the decoction was filtered before evaporated under reduced pressure to obtain a thick paste with a relative density of 1.27-1.30 (50℃), which was named extract S2.

Procedure 3:

S1 and S2 were mixed thoroughly with purified water and dextrin, and heated at constant temperature of 100℃ to evaporate excess water to refine the mixed solution into thick paste and extruded the thick paste into strips, rubbing pills to make wet pills, spheronizing, drying and screening into semi-finished products, and packing them into small packages to be Yi-Fei-San-Jie Pills (YFSJ).

As YFSJ has been used in the First Affiliated Hospital of Guangzhou University of Chinese Medicine as finished hospital preparations (Approval number of Guangdong province: Z20190015000) for more than 5 years, the production process was standardized and shown in patent (NO.201710010908.4) that can be searched on China National Intellectual Property Administration official website https://www.cnipa.gov.cn/. The net weight of each packet of pills is 8g, being equivalent to 16.84g of crude botanical drugs (seen as the annexed scanned PDF copy of the outer package).
